# *N*-3 Polyunsaturated Fatty Acids Improve Liver Lipid Oxidation-Related Enzyme Levels and Increased the Peroxisome Proliferator-Activated Receptor α Expression Level in Mice Subjected to Hemorrhagic Shock/Resuscitation

**DOI:** 10.3390/nu8040237

**Published:** 2016-04-22

**Authors:** Li Zhang, Feng Tian, Xuejin Gao, Xinying Wang, Chao Wu, Ning Li, Jieshou Li

**Affiliations:** 1 Department of General Surgery, Jinling Hospital, Medical School of Nanjing University, Zhongshan East Road 305, Nanjing 210002, China; zlshe1107@163.com (L.Z.); tianfeng_nju@163.com (F.T.); wuchao0008@126.com (C.W.); lingssh@126.com (N.L.); lijieshou1924@163.com (J.L.); 2 Department of General Surgery, Jinling Hospital, Clinical College of Southern Medical University, Zhongshan East Road 305, Nanjing 210002, China; xuejingao870214@163.com

**Keywords:** hemorrhagic shock, peroxisome proliferator-activated receptor, fatty acid oxidation, fish oil

## Abstract

Appropriate metabolic interventions after hemorrhagic shock/resuscitation injury have not yet been identified. We aimed to examine the effects of fish oil on lipid metabolic intervention after hemorrhagic shock/resuscitation. Firstly, 48 C57BL/6 mice were assigned to six groups (*n* = 8 per group). The sham group did not undergo surgery, while mice in the remaining groups were sacrificed 1–5 days after hemorrhagic shock/resuscitation. In the second part, mice were treated with saline or fish oil (*n* = 8 per group) five days after injury. We determined serum triglyceride levels and liver tissues were collected and prepared for qRT-PCR or Western blot analysis. We found that triglyceride levels were increased five days after hemorrhagic shock/resuscitation, but decreased after addition of fish oil. After injury, the protein and gene expression of carnitine palmitoyltransferase 1A, fatty acid transport protein 1, and peroxisome proliferator-activated receptor-α decreased significantly in liver tissue. In contrast, after treatment with fish oil, the expression levels of these targets increased compared with those in the saline group. The present results suggest *n*-3 polyunsaturated fatty acids could improve lipid oxidation-related enzymes in liver subjected to hemorrhagic shock/resuscitation. This function is possibly accomplished through activating the peroxisome proliferator-activated receptor-α pathway.

## 1. Introduction

Hemorrhagic shock (HS) following trauma is the second major cause of death among persons under the age of 45, accounting for 12.5%–26.6% of deaths in this age group [1]. Inadequate tissue perfusion in HS and subsequent reperfusion (HS/R) leads to gross metabolic disturbances and energy failure, which could lower the patients’ tolerance for traumatic stress, delay recovery, increase burden to organs, and even cause death [2].

Numerous studies have shown that substrate metabolism in critically ill patients is significantly altered when patients experience stress [3], particularly during the period of stress following trauma and shock [4]. In addition to hyperglycemia and negative nitrogen balance, increased lipid mobilization with the increase in free fatty acids (FFAs) and decreased lipogenesis are observed after stress. Moreover, the breakdown of peripheral fat, a process called lipolysis, is accelerated. Most of the FFAs released from adipose tissue undergo re-esterification in hepatic cells via the so-called triglyceride (TG)-FFA cycle, which has been shown to be accelerated during traumatic stress. Ectopic deposition of lipids in visceral organs, which may aggravate organ dysfunction, has also been observed during stress. However, few FFAs are used for oxidation, and this level is not sufficient to meet the body’s energy demands during stress [5,6]. Mitochondrial dysfunction in hepatic cells and muscle cells during thermal injury leads to decreased fat oxidation, further compounding this energy deficit [7]. However, few studies have examined changes in triglycerides and fat oxidation after HS/R.

During various types of stress, the expression levels of key enzymes involved in mitochondrial fatty acid oxidation, such as carnitine palmitoyltransferase 1A (CPT-1A) and fatty acid transport protein 1 (FATP-1), decrease significantly, resulting in substantial decreases in fatty acid oxidation. However, it is unclear how the expression levels of these key enzymes change during HS/R injury.

Peroxisome proliferator-activated receptors (PPARs) are a family of transcriptional factors. Among PPAR proteins, PPARα has been shown to be involved in lipid metabolism through upregulation of fatty acid oxidation [8]. Activated PPARs upregulate the expression of genes encoding enzymes involved in downstream lipid oxidation [9]. In our previous study, we found that *n*-3 polyunsaturated fatty acids (PUFAs) could activate PPAR signaling [10]. Additionally, researchers have suggested that *n*-3 PUFAs, which are abundant in fish oil, regulate lipid metabolism [11]. Thus, *n*-3 PUFAs promote lipolysis and fatty acid oxidation and inhibit lipogenesis.

Therefore, in this study, we aimed to examine the effects of fish oil on lipid metabolic intervention after HS/R. We hypothesized that HS/R may increase triglyceride levels in the blood by suppressing fat oxidation and that *n*-3 polyunsaturated fatty acids could reverse this effect.

## 2. Materials and Methods

### 2.1. Animals

Animal studies were carried out in a specific pathogen-free facility accredited by the Association for Assessment and Accreditation of Laboratory Animal Care International, and all animal protocols were approved by the Animal Care and Use Committee of the Model Animal Research Center (NIH publication No. 86–23, revised 1985), the host for the National Resource Center for Mutant Mice (Nanjing University, China). Adult male C57BL/6 mice (10–12 weeks old, weighing 25–30 g each) were purchased from the National Resource Center for Mutant Mice. The mice were housed in plastic cages (five animals/cage) at a constant temperature of approximately 22 °C (20–26 °C) and 40%–70% relative humidity with a 12-h:12-h light:dark cycle and fed a standard laboratory mouse diet and water *ad libitum*. The mice were allowed to acclimate to their new environment for at least 7 days prior to manipulation. One day prior to surgery, mice were placed on a pre-operative complete liquid diet. Every effort was made to minimize animal number and suffering during the experiments.

### 2.2. Animal Treatments and Groups

The experiment was divided into two parts. In Part 1, 48 C57/BL6 mice were divided into six groups (*n* = 8 mice per group). In the sham group, animals were subjected to 1.5 h of anesthesia and 2 h of recovery before euthanasia to obtain physiological baseline levels. Mice in the remaining five groups were subjected to HS/R injury and then sacrificed at 1–5 days after HS/R. In Part 2, 24 C57BL/6 mice were assigned to three groups (*n* = 8 mice per group). Sham group was the same as that in Part 1. Mice in FO or Saline group were subjected to femoral artery catheterization, 1.5 h of anesthesia with pressure-controlled HS followed by administration of either saline (0.2 mL/kg body weight) or fish oil (0.2 g/kg weight) through the caudal vena cava once per day for 5 days after injury.

### 2.3. HS Model

Mice were anesthetized by intraperitoneal administration of 0.15 mL/10 g body weight of 2.5% avertin and subcutaneous administration of 5 mg/kg body weight carprofen. The right and left femoral arteries were cannulated with PE10 tubing, and the right arterial catheter was connected to a pressure monitor to measure the mean arterial pressure (MAP). Small bowel resection was performed by transecting the small bowel in two places at 3 cm distal to Treitz’s ligament and removing 2 cm of jejunum. Intestinal continuity was subsequently restored with an end-to-end, single-layered anastomosis using 9–0 monofilament sutures. The abdominal cavity was then closed with 4–0 continuous sutures. Before closing the abdomen, 0.1 mL cefuroxine (20 mg/kg) was administered into the abdominal cavity to prevent infection. Next, blood was withdrawn over 15 min via the left femoral artery catheter to reduce the MAP to 35 mmHg. Blood was withdrawn and returned to the animal as needed to maintain a MAP of 35 ± 5 mmHg. At the end of the shock period (90 min), the mice were resuscitated using the shed blood plus two volumes of Ringer’s lactate. The catheter was removed, the artery was ligated, and the skin incision was closed. During the surgery and recovery, the mice were placed on heating platform. When there was any evidence of struggle, shortness of breath, or any other reactions during the surgery, mice were further anesthetized by intraperitoneal administration of 0.15 mL/10 g body weight of 2.5% avertin. Medical monitoring was performed during recovery from surgery and anesthesia. Carprofen (4 mg/kg) was injected subcutaneously as the animals began physical activity and to block pain or distress during recovery. Carprofen was also administered at 12 and 24 h after surgery for pain control.

### 2.4. Blood and Tissue Collection

Blood samples collected from the heart were immediately centrifuged (3000× *g* for 15 min at 4 °C), frozen under liquid nitrogen, and stored at −80 °C until further analysis. The liver was excised, dried with lint, and weighed. Small pieces of the larger lobe were frozen under liquid nitrogen and stored at −80 °C until further analysis. We determined serum TG levels on a HITACHI 7020 Biochemical Analyzer. We also determined serum cytokines level such as TNF-alpha, IL-1 and IL-6 using ELISA kit (ExCell Bio, Shanghai, China).

### 2.5. Western Blot Analysis

Whole-cell lysates from frozen tissues were isolated using RIPA lysis buffer (150 mM Tris-HCl, 50 mM·NaCl, 1% NP-40, 0.1% Tween-20). Protease and phosphatase inhibitors were added to all buffers before experiments. Protein concentrations were assayed using a BCA protein assay (Pierce, Rockford, IL, USA). Proteins were separated on polyacrylamide gels and transferred to membranes. After blocking nonspecific bindings, membranes were incubated with primary antibodies (anti-FATP-1, anti-CPT-1A, or anti-PPARα antibodies) overnight at 4 °C, followed by incubation with appropriate secondary antibodies. Specific proteins were then visualized by using ECL Plus reagents (Amersham Biosciences, New York, NY, USA). Band intensities were measured using ImageQuant (Bio-Rad Laboratories, Berkeley, CA, USA) and normalized to the expression of GAPDH.

### 2.6. Quantitative Real-Time Reverse Transcription Polymerase Chain Reaction (qRT-PCR)

Total RNA was extracted using TRIzol (Invitrogen, Carlsbad, CA, USA), and random hexamers were used to prime reverse-transcription reactions with Superscript III (Invitrogen). qRT-PCR was performed using an ABI 7300 detection system (Applied Biosystems, Foster City, CA, USA) with SYBR green I reagents (Takara, Shiga, Japan). PCR products were subjected to a melting curve analysis. At a specific threshold in the linear amplification stage, the cycle differences between amplified *36B4* (as an internal control) and cDNAs of *36B4* were used to determine the relative expression levels of *36B4*. Primer sequences used in this study are available on request (Table 1).

### 2.7. Statistical Analyzes

Data are expressed as means ± standard deviations (SDs). Student’s *t*-tests were used for direct comparisons between the means of two groups. First, Mauchly’s test of Sphericity was carried out to determine whether there were correlations between these repeated measurements at different time points. If the significance level of Mauchly’s test was not less than 0.05, then the Huynh–Feldt condition was met. Then Analysis of variance (ANOVA) was used for multiple comparisons between groups. Differences with *p* values of less than 0.05 were considered significant.

## 3. Results

### 3.1. TG Levels in Mice Subjected to HS/R

First, we measured TG levels in each group (Table 2 and Table 3). On Day 1 after HS/R injury, TG levels were similar to those in normal mice in the sham group (0.30 ± 0.07 *vs.* 0.32 ± 0.09, respectively; *p* = 0.699). However, on Day 5 after HS/R, TG levels were substantially increased compared with those in the sham control group (0.51 ± 0.13 *vs.* 0.32 ± 0.09, respectively; *p* = 0.001). Moreover, TG levels were significantly decreased following treatment with fish oil compared with those in the saline group (0.39 ± 0.07 *vs.* 0.47 ± 0.11, respectively; *p* = 0.048), reaching a level similar to that of the sham group (*p* = 0.240).

### 3.2. Changes in Serum Pro-Inflammation Cytokines Level in Mice Subjected to HS/R

We also determined the serum pro-inflammation cytokines levels in each group (Table 4 and Table 5). During Day 1 to Day 5 after HS/R injury, the level of TNF-α did not change significantly, which was similar to that in normal mice in the sham group. There was no significant difference in the levels of IL-1 and IL-6 after HS/R injury among all the groups. Moreover, the levels of these serum pro-inflammation cytokines did not change significantly following treatment with fish oil compared with those in the saline group and in the sham group.

### 3.3. Changes in CPT-1A Expression in the Liver

**Figure 1:**
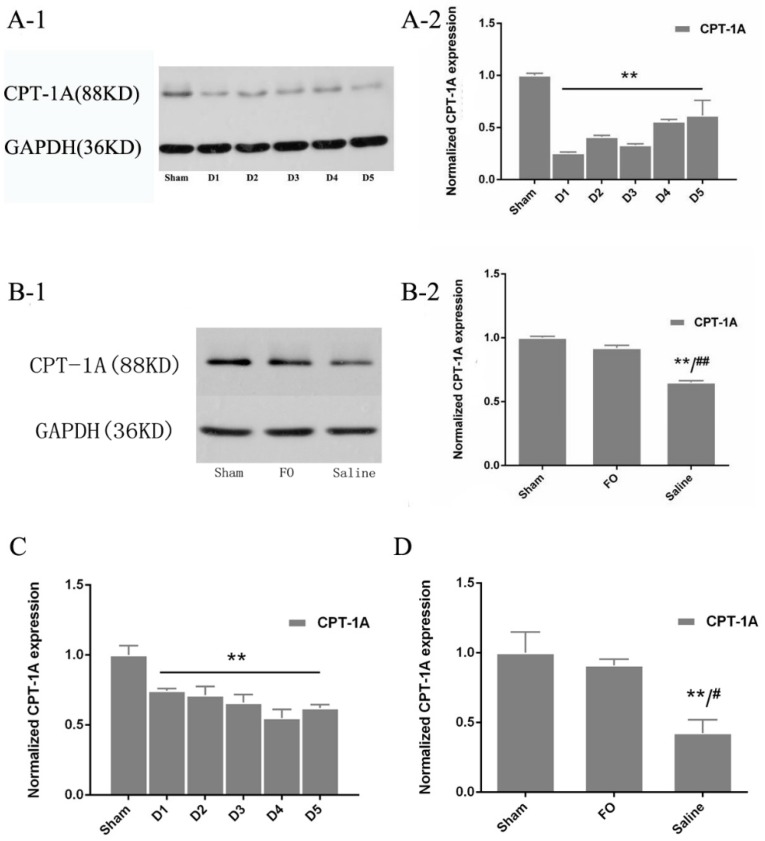
Western blot analysis and qPCR analysis of CPT-1A expression: (**A-1**) Western blots of CPT-1A in the Part 1 experiment; (**A-2**) the relative expression of CPT-1A protein was quantified and normalized to that of GAPDH protein; (**B-1**) Western blots of CPT-1A in the Part 2 experiment; (**B-2**) the relative expression of CPT-1A protein was quantified and normalized to that of GAPDH protein; (**C**) the relative expression of CPT-1A was normalized to that of 36B4 in the Part 1 experiment; and (**D**) the relative expression of CPT-1A was normalized to that of 36B4 in the Part 2 experiment. * *p* < 0.05 compared to the sham group; ** *p* < 0.01 compared to the sham group; ^#^ *p* < 0.05 compared to the FO group. Error bars indicate standard deviations. D1–D5 indicate days after operation in the experimental groups.

**Figure 2:**
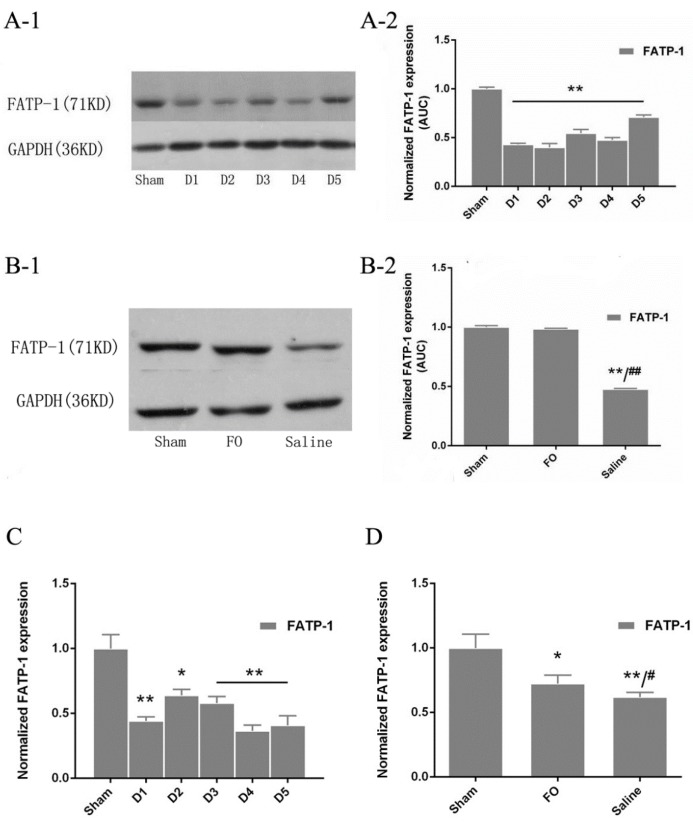
Western blot analysis and qPCR analysis of FATP-1 expression: (**A-1**) Western blots of FATP-1 in the Part 1 experiment; (**A-2**) the relative expression of FATP-1 protein was quantified and normalized to that of GAPDH protein; (**B-1**) Western blots of FATP-1 in the Part 2 experiment; (**B-2**) the relative expression of FATP-1 protein was quantified and normalized to that of GAPDH protein; (**C**) the relative expression of FATP-1 was normalized to that of 36B4 in the Part 1 experiment; and (**D**) the relative expression of FATP-1 was normalized to that of 36B4 in the Part 2 experiment. * *p* < 0.05 compared to the sham group; ** *p* < 0.01 compared to the sham group; ^#^ *p* < 0.05 compared to the FO group. Error bars indicate standard deviations. D1–D5 indicate days after operation in the experimental groups.

On Day 1 after HS/R injury, the relative expression of CPT-1A protein was significantly reduced compared with that in the sham group (25.4% *vs.* 100%, respectively; *p* < 0.01; Figure 1A-1,A-2). Consistent with these changes in protein expression, qPCR analysis showed that *CPT-1A* mRNA expression was significantly reduced after injury compared with that in the sham group (Figure 1C). Moreover, the addition of fish oil significantly increased the expression of CPT-1A in liver tissues compared with that in the saline group (92.0% *vs.* 64.9%, respectively; *p* < 0.01), and the expression of CPT-1A in mice treated with fish oil was statistically similar to that in the sham group (*p* > 0.05; Figure 1B-1,B-2). Administration of fish oil also significantly increased the expression of *CPT-1A* mRNA (Figure 1D).

### 3.4. Changes in FATP-1 Expression in the Liver

FATP-1 protein expression was significantly decreased after HS/R injury as compared with that in the sham group (Figure 2A-1,A-2). Consistent with these changes in protein expression, *FATP-1* mRNA expression was also reduced following HS/R injury (Figure 2C). The addition of fish oil significantly enhanced the expression of FATP-1 protein in the liver as compared with that in the saline group, and the level of tissue FATP-1 protein recovered to levels similar to those in the sham group after treatment with fish oil (fish oil group: 98.5% *versus* sham group: 100%; *p* = 0 .37; Figure 2B-1,B-2). Moreover, supplementation with fish oil significantly increased the relative expression of *FATP-1* mRNA compared with that in the saline group (72.4% *versus* 62.0%, respectively; *p* < 0.05). The expression of *FATP-1* mRNA in mice treated with fish oil remained lower than that in the sham group (Figure 2D).

### 3.5. Changes in PPAR-α Expression in the Liver

**Figure 3:**
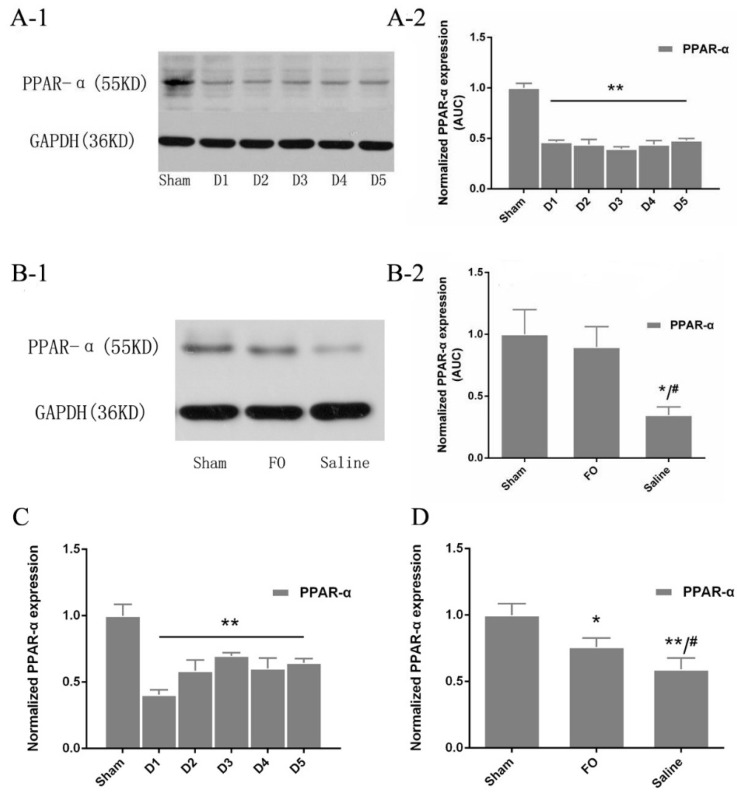
Western blot analysis and q-PCR analysis of PPAR-α expression: (**A-1**) Western blots of PPAR-α in the Part 1 experiment; (**A-2**) the relative expression of PPAR-α protein was quantified and normalized to that of GAPDH protein; (**B-1**) Western blots of PPAR-α in the Part 2 experiment; (**B-2**) the relative expression of PPAR-α protein was quantified and normalized to that of GAPDH protein; (**C**) the relative expression of PPAR-α expression was normalized to that of 36B4 in the Part 1 experiment; and (**D**) the relative expression of PPAR-α expression was normalized to that of 36B4 in the Part 2 experiment. * *p* < 0.05 compared to the sham group; ** *p* < 0.01 compared to the sham group; ^#^ *p* < 0.05 compared to the FO group. Error bars indicate standard deviations. D1–D5 indicate days after operation in the experimental groups.

PPAR-α protein was significantly downregulated in liver tissues after HS/R compared with that in the sham group (Figure 3A-1,A-2). Consistent with these changes in protein expression, the level of *PPAR-α* mRNA was also lower after HS/R than that in the sham group (Figure 3C). The overall expression of PPAR-α was increased after five days of fish oil supplementation compared with that in the saline group (74.3% *vs.* 56.8%, respectively; *p* = 0.038; Figure 3B-1,B-2). Similarly, the addition of fish oil significantly increased the expression of *PPAR-α* mRNA compared with that in the saline group, but *PPAR-α* mRNA expression in mice treated with fish oil was still significantly lower than that in the sham group (Figure 3D).

## 4. Discussion

In this study, we examined the effects of fish oil on lipid metabolism after HS/R. Overall, our data suggested that *n*-3 polyunsaturated fatty acids could improve liver lipid oxidation and increased the PPAR-α expression in mice subjected to HS/R. This function is possibly accomplished through activating the peroxisome proliferator-activated receptor-α pathway. Thus, these data have important implications in the prevention of complications after HS/R in the context of traumatic stress.

Ischemia/reperfusion (I/R) results in a variety of cellular metabolic processes that ultimately lead to tissue and organ dysfunction or failure. Hypoperfusion of tissues decreases the removal of waste products and the supply of oxygen and nutrients, further aggravating cellular dysfunction. Postinjury cellular dysfunction and hormone responses appear to be associated with multiple metabolic abnormalities, notably, significant changes in fat metabolism [12], leading to insufficient energy supply and metabolic disorders. While lipolysis is known to be increased after severe stress, fatty acid oxidative ability remains variable. For example, in a study by Cree *et al.* [13], human mitochondrial fatty acid oxidative capacity is acutely impaired following burn trauma. Takeyama and Beylot discovered that hepatic fatty acid oxidation is suppressed in endotoxic rats and patients with sepsis [14,15]. However, Nordenstrom *et al.* [16] found that trauma was followed by an increase in the oxidation rate of exogenous fat during total parental nutrition. In the present study, we examined the liver fatty acid oxidative capacity after HS/R by measuring the expression levels of two rate-limiting enzymes for fatty acid beta-oxidation. Our data showed that HS/R impaired liver fatty acid oxidation from Days 1–5 after injury. Thus, the oxidation of fatty acids was inadequate to meet energy needs, consistent with the results of multiply studies of severe injury [5,17].

CPT-1A is the rate-limiting enzyme for mitochondrial fatty acid oxidation. After conversion of fatty acids into fatty acyl-CoA, mitochondrial CPT-1A metabolizes fatty acyl-CoA into acylcarnitine. In this study, the protein and mRNA expression of CPT-1A in the liver was significantly depressed after HS/R injury, indicating that HS/R injury impaired fatty acid oxidation in the liver. These results are generally consistent with those of a previous study on shock [18]. Additionally, hypoxia induces suppression of fatty acid β-oxidation, with a significant reduction in CPT-1A mRNA expression [19]. In rats, substantial burn causes a decrease in the expression of the *CPT-1A* gene, and genes involved in protein production and activation within skeletal muscle are downregulated at one and three days post-burn in rats [20,21]. However, changes in the expression of CPT-1A in the liver during sepsis are not clear. In a previous study, lipopolysaccharide (LPS) was shown to decrease fatty acid oxidation and the expression of CPT-1A, a key protein required for fatty acid oxidation in the diaphragm [22]. We observed, for the first time, a decrease in CPT-1A expression after HS/R injury, consistent with the findings from previous studies of other types of stress.

In this study, we also measured the expression of FATP-1 in liver tissues after HS/R injury. Membrane-associated FATPs are required for fat uptake and transport fatty acids to the mitochondria for oxidation, which is suppressed during the acute phase response. LPS and cytokines differentially regulate the hepatic mRNA expression of FATPs [23]. The decrease in FATP expression may also contribute to decreased fatty acid oxidation during infection. Indeed, LPS has also been shown to decrease the expression of FATP-1 in the diaphragm [22]. Consistent with these previous reports, we found that FATP-1 expression was suppressed after HS/R in mice. Therefore, fish oil could increase the levels of fatty acid oxidation-related enzymes after HS/R injury, which may regulate substrate metabolism and decrease metabolic complications.

We also observed a significant increase in TG levels on Day 2 after HS/R injury, with the most dramatic increase observed on Day 5. Increased TG levels result from metabolic alterations that increase TG synthesis, decrease TG clearance, or both. FFAs are normally released from adipose tissue after hemorrhagic trauma in quantities that exceed what is used for oxidation. According to our results, increased TG levels could be explained by decreased expression of oxidative enzymes, such as CPT-1A and FATP-1, after HS/R injury. The excess levels of TGs are taken up by adipose tissue and re-esterified to TGs in a process called the TG-FAA cycle [5]. This continuous cycle is considered futile, because the released FFAs are not used for energy production but rather continue to be recycled again into TGs. Consistent with our study, hypertriglyceridemia commonly occurs during sepsis, and serum TG levels are increased during infection/inflammation by multiple cytokines in rodents and humans [24,25]. The hypertriglyceridemic effect of LPS and cytokines is rapid, occurring within 2 h after administration, and is sustained for at least 24 h [26,27]. In a study of 182 patients with systemic inflammatory response syndrome/severe sepsis or shock, patients with shock were found to have higher TG and FFA levels than patients without shock [20] due to reduced liver oxidation and increased adipose tissue lipolysis [28]. We might be able to use fish oil to correct the hypertriglyceridemia occurring after HS/R injury.

Recent studies have shown that *n*-3 PUFAs rich in fish oil regulate lipid metabolism [11], promoting lipolysis and fatty acid oxidation and inhibiting lipogenesis. Interestingly, we found that administration of fish oil significantly decreased TG levels compared with administration of saline. Moreover, addition of fish oil significantly improved the expression of CPT-1A and FATP-1 in the liver after shock. These changes in fatty acid metabolism after HS/R injury are likely mediated by changes in the levels of nuclear hormone receptors, which regulate the expression of enzymes and transport proteins involved in fatty acid oxidation. Fenofibrate, a PPAR-α agonist, has been shown to have a positive influence on oxidative enzymes, such as CPT-1A, in the liver and adipose tissue [29]. In the present study, we demonstrated that protein and mRNA levels of PPAR-α were decreased in mice subjected to HS/R, but recovered following administration of fish oil. *N*-3 PUFAs might reduce pro-inflammation cytokines, which is known to suppress the expression of many genes involived in fatty acid oxidation in the liver, including PPAR-α [30]. We determined cytokines such as TNF-alpha, IL-1 and IL-6 from Day 1 to Day 5 after injury in this study. We found that the levels of these cytokines did not have the significant difference during these days. They nearly got back to normal level over 24 h after the HS/R. Moreover, the cytokines levels are similar between the treatment groups. Thus, we do not have the positive evidence that the increase expression of PPARα is related to the anti-inflammatory effect of fish oil in this situation. Additionally, *n*-3 PUFA as a ligand for PPAR-α has also been shown to activate PPAR-α [31]. Thus, our results showed that fish oil may improve fat β-oxidation after HS/R injury by triggering PPAR-α expression. 

There are still several limitations to our present study. First, we only examined the levels of TGs and did not measure the fatty acid oxidation rate after HS/R. Plasma TG levels are an indirect index of lipid oxidation. Moreover, while we monitored indirect calorimetry, we found that mice after injury had significantly more oxygen consumption, more carbon dioxide emission, and increased energy expenditure (data shown in Supplementary Materials Figure S1); however, this is still indirect evidence. Additionally, we only evaluated the expression levels of enzymes and protein involved in the uptake and oxidation of fatty acids. Thus, future studies are needed to evaluate the expression of enzymes related to TG synthesis, which could influence TG levels. Finally, we did not measure PPAR-α activity neither use pharmacologic, so we do not have the direct evidence presenting the link between the PPARa activity and the observed phenotype with TAG, CPT1 and FATP after lipid infusion. Therefore, we plan to examine PPAR-α activity and determine the effects of fish oil in the presence of PPAR-α agonists and inhibitors in subsequent studies.

## 5. Conclusions

In conclusion, we found that HS/R injury could increase plasma TG levels and lead to disruption of lipid metabolism in the liver. Enzymes and transport proteins involved in fatty acid oxidation were downregulated after HS/R injury, concomitant with the suppression of PPAR expression. Our data also showed that *n*-3 PUFA supplementation improved these disruptions, possibly through the effects of PPAR-α on lipid metabolism. Thus, the results of our study indicated that *n*-3 PUFA supplementation might be a novel strategy for regulation of lipid oxidation in the liver during HS/R injury.

## Figures and Tables

**Table 1:** Primer sequences.

Primer	Primer Sequences (5′to3′)
FATP1 Forward	TCTGTTCTGATTCGTGTTCGG
FATP1 Reverse	CAGCATATACCACTACTGGCG
CPT-1A Forward	CTATGCGCTACTCGCTGAAGG
CPT-1A Reverse	GGCTTTCGACCCGAGAAGA
PPAR-α Forward	TACTGCCGTTTTCACAAGTGC
PPAR-α Reverse	AGGTCGTGTTCACAGGTAAGA
36B4 (internal control) Forward	TGAGATTCGGGATATGCTGTTGG
36B4 (internal control) Reverse	CGGGTCCTAGACCAGTGTTCT

**Table 2:** The level of TG in different groups in Part 1.

Group	TG (mmol/L)	*p*
Sham	0.32 ± 0.09	
D1	0.30 ± 0.07	0.699
D2	0.39 ± 0.08	0.210
D3	0.42 ± 0.09	0.055
D4	0.44 ± 0.11	0.024 *
D5	0.51 ± 0.13	00.001 **

Notes: *p*: compared with Sham group, *: *p* < 0.05 and **: *p* < 0.01.

**Table 3:** The level of TG in different groups in Part 2.

Group	TG (mmol/L)	*p*
Sham	0.34 ± 0.19	
FO	0.39 ± 0.07	0.240
Saline	0.47 ± 0.11	0.048 *

Notes: *p*: compared with Sham group; *: *p* < 0.05.

**Table 4:** The level of serum cytokines in different groups in Part 1.

Group	TNF-α (pg/mL)	IL-1 (pg/mL)	IL-6 (pg/mL)
Sham	22.01 ± 9.14	10.10 ± 7.23	13.00 ± 10.23
D1	23.66 ± 13.44	12.14 ± 7.67	11.75 ± 8.06
D2	26.15 ± 10.67	14.33 ± 13.04	19.75 ± 12.95
D3	22.06 ± 6.63	12.37 ± 7.49	13.25 ± 7.68
D4	22.18 ± 7.48	11.43 ± 8.03	10.25 ± 7.93
D5	24.38 ± 10.19	10.95 ± 8.98	14.25 ± 9.29

**Table 5:** The level of serum cytokines in different groups in Part 2.

Group	TNF-α (pg/mL)	IL-1 (pg/mL)	IL-6 (pg/mL)
Sham	23.97 ± 9.35	10.18 ± 7.42	13.12 ± 9.73
FO	23.66 ± 13.44	12.14 ± 7.67	11.75 ± 8.06
Saline	26.15 ± 10.67	14.33 ± 13.04	13.25 ± 12.95

## Supplementary Materials

The following are available online at http://www.mdpi.com/2072-6643/8/4/237/s1. Figure S1: Energy expenditure of sham group and D5 group mice. We monitored the energy expenditure by indirect calorimetry, a comprehensive lab animal monitoring system (Columbus Instruments, Columbus, OH, USA). Volume of O_2_ consumption and CO_2_ production were continuously recorded over 72-h period. Respiratory exchange ratio (RER) was calculated as the molar ratio of VCO_2_:VO_2_. Heat was calculated as Heat = (3.815 + 1.232 * RER) * VO_2_ (Figure S1).
