# Supplementary material for: N-3 Polyunsaturated Fatty Acids Improve Liver Lipid Oxidation-Related Enzyme Levels and Increased the Peroxisome Proliferator-Activated Receptor α Expression Level in Mice Subjected to Hemorrhagic Shock/Resuscitation
Source: Nutrients. 2016 Apr 22;8(4):237. doi: 10.3390/nu8040237 (PMC4848705; doi:10.3390/nu8040237)
Supplement: Supplementary file 1 [file nutrients-08-00237-s001.docx]

**Supplementary Materials: *N*-3 Polyunsaturated Fatty Acids Improve Liver Lipid Oxidation-related Enzyme Levels and Increased the Peroxisome Proliferator-Activated Receptor α Expression Level in Mice Subjected to Hemorrhagic Shock/Resuscitation**

Li Zhang, Feng Tian, Xuejin Gao, Xinying Wang, Chao Wu, Ning Li and Jieshou Li


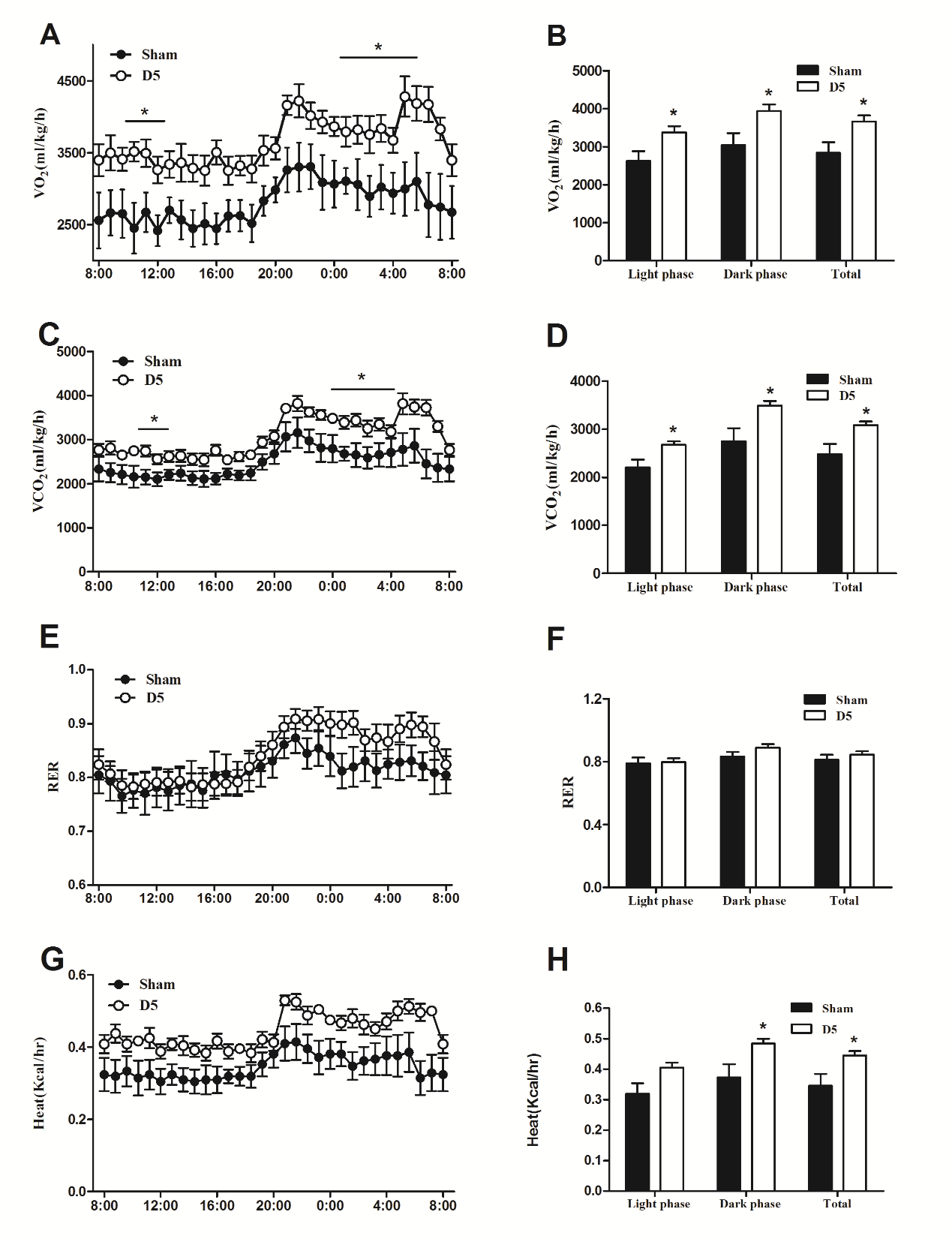


**Figure S1.** Energy expenditure of sham group and D5 group mice. (**A**,**B**) Volume of O_2_ consumption; (**C**,**D**) Volume of CO_2_ production; (**E**,**F**) RER and (**G**,**H**) Heat. * *p* < 0.05 compared to the sham group; Error bars indicate standard deviations. D5 indicate days after operation in the experimental groups.
